# Transcriptome assembly and annotation of johnsongrass (*Sorghum halepense*) rhizomes identify candidate rhizome‐specific genes

**DOI:** 10.1002/pld3.65

**Published:** 2018-06-19

**Authors:** Nathan Ryder, Kevin M. Dorn, Mark Huitsing, Micah Adams, Jeff Ploegstra, Lee DeHaan, Steve Larson, Nathan L. Tintle

**Affiliations:** ^1^ Departments of Biology and Statistics Dordt College Sioux Center Iowa; ^2^ Department of Plant Pathology Kansas State University Manhattan Kansas; ^3^ The Land Institute Salina Kansas; ^4^ USDA‐ARS, Forage and Range Research Laboratory Utah State University Logan Utah

**Keywords:** next‐generation sequencing, perennial plants, rhizomes, RNA‐Seq, *Sorghum halepense*, transcriptomics/transcriptome analysis

## Abstract

Rhizomes facilitate the wintering and vegetative propagation of many perennial grasses. *Sorghum halepense* (johnsongrass) is an aggressive perennial grass that relies on a robust rhizome system to persist through winters and reproduce asexually from its rootstock nodes. This study aimed to sequence and assemble expressed transcripts within the johnsongrass rhizome. A de novo transcriptome assembly was generated from a single johnsongrass rhizome meristem tissue sample. A total of 141,176 probable protein‐coding sequences from the assembly were identified and assigned gene ontology terms using Blast2GO. Estimated expression analysis and BLAST results were used to reduce the assembly to 64,447 high‐confidence sequences. The johnsongrass assembly was compared to *Sorghum bicolor*, a related nonrhizomatous species, along with an assembly of similar rhizome tissue from the perennial grain crop *Thinopyrum intermedium*. The presence/absence analysis yielded a set of 98 expressed johnsongrass contigs that are likely associated with rhizome development.

## 1 INTRODUCTION

Rhizomes are the horizontally aligned subterranean stems which allow a perennial plant to grow back from dormancy after a period of harsh seasonal conditions. With rhizomes at sufficient depth, an herbaceous plant may reemerge each year without having to make the extensive investment into root growth required by germination. The growing season for a rhizomatous plant is lengthened when its roots reach below the top layer of the soil, reducing the effects of environmental stresses such as temperature. Furthermore, a large persisting root network allows perennial plants to limit soil erosion, reduce runoff, and store more carbon underground as compared to an annual crop (Cox, Glover, Van Tassel, Cox, & DeHaan, 2006).

Annual species such as wheat or corn require frequent disruptions from tillage and can only achieve diminished root depth and length before harvest. Soils planted with annual crops are then susceptible to excessive erosion. A 100‐year agricultural experiment revealed that plots with continuously cropped corn retained less than half the amount of topsoil as plots with only perennial grasses (Gantzer, Anderson, Thompson, & Brown, 1990). Annual root systems also exacerbate nutrient runoff. Corn, wheat, and rice have shown nitrogen fertilizer uptake efficiencies ranging from 18 to only 49% (Cassman, Dobermann, & Walters, 2002). In fact, the loss of nitrate N through subsurface drainage may be 30 to 50 times greater in annual than in perennial crops (Randall & Mulla, 2001).

Perennial plants present further ecological advantages via carbon sequestration. Typical annual crops increase soil organic carbon by 0–30 $\frac{g}{m^{2}}$ in a year, while perennials plants were found to accumulate 32–44 $\frac{g}{m^{2}}$ (Robertson, Paul, & Harwood, 2000). Perennial crops not only store more carbon, but also have no requirement to expend fossil fuels on tillage. They have a negative global warming potential, in CO_2_ equivalents, while annual crops actually increase atmospheric carbon (Robertson et al., 2000).

*Sorghum halepense* is a perennial grass which has been classified as an invasive weed in 53 countries (Holm, Plucknett, Pancho, & Herberger, 1977). *Sorghum halepense* rhizomes, which are known to regenerate even when cut into pieces, allow the plant to endure winters and extend locally (Howard, 2004). One plant may grow 275 feet of rhizomes in a single growing season. Moreover, johnsongrass is self‐fertile with a high rate of seed production, yielding more than 80,000 seeds in the same growing season (Howard, 2004; Johnson, Kendig, Smeda, & Fishel, 1997). This propensity for reproduction, coupled with rapid growth relative to native grasses, can give johnsongrass a competitive edge and lead to monocultures in unsupervised areas. Johnsongrass also exhibits the ability to grow in a wide range of environmental conditions and has resistances to many common herbicides and pathogens (Howard, 2004; Johnson et al., 1997). With such vigorous root systems and high fecundity, this species is an excellent model for understanding one important strategy of the perennial growth habit. Sequences that are expressed specifically in Johnsongrass rhizomes and found to be related to resource management or asexual reproduction could improve prediction of perennial behavior in perennial grasses (Jang et al., 2009).

Attempts at breeding a perennial hybrid from diploid *S. bicolor* and tetraploid *S. halepense* began in earnest before 2003 at the Land Institute in Salina, Kansas (Cox et al., 2006). Developing a hybrid with sufficient yield may require an extensive time‐frame, but this can be expedited through the use of genetically informed techniques. Marker‐assisted selection could make use of QTLs discovered in johnsongrass that are linked to rhizome activity (Xiong et al., 2007).

Previous studies have aligned johnsongrass RNA transcripts with QTLs likely related to rhizome function in sorghum and rice (Jang et al., 2006, 2009), but lack the sheer quantity of data expected from sequencing technologies today. This study provides a large, novel transcriptome for rhizome tissue from johnsongrass. The sequenced rhizome RNA was assembled, filtered, and annotated with up‐to‐date tools for nonmodel organisms. An annotated transcriptome for johnsongrass rhizomes then facilitates the discovery of additional rhizome‐enriched transcripts. Further analysis with BLAST compared assembled johnsongrass sequences with nonrhizomatous and rhizomatous species and has led to a reduced set of candidate genes for rhizome‐related function.

## 2 MATERIALS AND METHODS

### 2.1 Plant origin and RNA extraction

Johnsongrass plants were harvested at the coordinates 38°46′15.46″ N and 97°34′21.77″ W near Salina, Kansas on July 7, 2015. They were shipped overnight on dry ice to Sioux Center, Iowa, where the rhizome apical meristems were removed, immersed in liquid nitrogen, and placed in a −80^o^C freezer. RNA was extracted from four samples of rhizome node buds using the RNeasy Plant Mini Kit (Qiagen) precisely following recommended protocols. DNase digestion was then performed with the TURBO DNA‐*free* kit (Thermo Fisher Scientific), followed by the RNA cleanup procedure from the RNeasy mini handbook. Each sample received an A_260_/A_280_ ratio greater than 2 in a NanoDrop 2000 Spectrophotometer (Thermo Scientific). All four samples were then sent to the University of Minnesota Genomics Center for sequencing.

*Thinopyrum intermedium* rhizome tissue was obtained from a clone of a plant derived from The Land Institute's breeding program (C3‐3471). RNA was extracted as described above.

### 2.2 Library construction and next‐generation sequencing

The four johnsongrass samples were run through a denaturing agarose gel to visualize RNA integrity (Supporting Information Figure S1). Samples 3 and 4, which depicted intact RNA, were pooled and submitted for library prep with the TruSeq Stranded mRNA Library Prep kit (Illumina). The resulting cDNA library was sequenced in a single lane of High Output (2 × 125 bp) on the Illumina HiSeq 2500 platform. The raw reads were trimmed in BBduk (Bushnell, 2014), removing adaptors and reads that were short or low quality.

*Thinopyrum intermedium* rhizome RNA libraries were prepared using the Illumina TruSeq RNA Library Prep Kit v2 by the University of Minnesota Genomics Center and sequenced across three lanes of an Illumina HiSeq 2000 (2 × 100 bp) run. Libraries were size‐selected with a Caliper LabChip XT (PerkinElmer) to have an approximate insert size of 200 bp. The resulting reads were also trimmed with bbduk to produce 5.7 gigabases of high‐quality (>Q30) data as 65,413,146 paired‐end reads (NCBI Sequence Read Archive SRX3529031).

### 2.3 Trinity assembly and TransDecoder identification of protein‐coding sequences

The cleaned reads from each organism were de novo assembled in Trinity (version 2.4.0) (Grabherr et al., 2011; Haas et al., 2013). The johnsongrass assembly was run through TransDecoder (version 3.0.1), a companion to Trinity which uses open reading frames within assembly transcripts to find likely coding regions (Haas & Papanicolaou, 2017).

### 2.4 Annotation with Blast2GO PRO

The predicted coding sequences from TransDecoder were annotated in Blast2GO PRO, an all‐in‐one tool that befits large‐scale functional annotation with Gene Ontology (GO), especially for an original assembly of a nonmodel organism. The suite performs the BLAST algorithm to align FASTA‐formatted sequence inputs with homologs in specified databases. Annotations are mapped to query sequences by their association with BLAST hits (Conesa et al., 2005).

The BLASTX‐fast search was used, aligning the coding sequences against the NCBI nonredundant protein database (NR). Query sequences with BLAST hits were mapped to GO terms and assigned functional annotations where available. See Supporting Information Data S2 for Blast2GO results on the TransDecoder predicted coding sequences.

### 2.5 Estimated transcript abundance with kallisto

To produce approximate abundances of the assembly transcripts, the quantification software kallisto (version 0.44.0) was used via the Trinity toolkit. Kallisto makes an index and performs pseudoalignment to quickly quantify the expression of a set of transcripts, while maintaining similar or greater accuracy than existing methods (Bray, Pimentel, Melsted, & Pachter, 2016). See Supporting Information Data S1 for the top 20 most expressed *S. halepense* sequences from kallisto with descriptions and annotations.

### 2.6 Assembly filtering

The johnsongrass assembly was further reduced by filtration of estimated expression levels and top BLAST hit organisms. All lowly expressed transcripts, having a FPKM (fragments per kilobase transcript per million) less than 1, were removed from the assembly as possible background transcription (Davidson et al., 2012). This threshold may have also excluded truly expressed sequences, but was supported by the distribution of estimated abundances (Supporting Information Figure S2). All transcripts with a top BLAST hit that was not a plant were also removed, being potential contaminants. The final, reduced assembly then contained sequences that were very likely to be truly expressed and very unlikely to be from contaminant reads.

### 2.7 Comparative transcriptomics with *S. bicolor* and *Th. intermedium* rhizomes

Two sequenced genomes closely related to *S. halepense* were selected: *S. bicolor* (a close annual relative and possible ancestor of *S. halepense* (Jang et al., 2009)) and *Th. intermedium* (a recently sequenced rhizomatous perennial grass). The JGI Plant Flagship genome of nonrhizomatous *S. bicolor* (version 3.1.1) was accessed from the NCBI RefSeq database (Accession ID: ABXC03000000). The protein model reference sequences were formatted into a protein BLAST database. Predicted transcripts for the rhizomatous species *Th. intermedium* were obtained from a prepublication annotated assembly (JGI annotation version 2.1) (Dorn et al., unpublished—pending release onto Phytozome) and were formatted into a second protein BLAST database for comparison.

Using the *S. halepense* proposed protein‐coding sequences as queries, two BLASTX searches were performed against the custom protein databases of *Th. intermedium* and *S. bicolor*. The query sequences that received one or more BLAST hit (e‐value < 10^−20^) were cross‐listed between the two searches, and removed. The *S. halepense* sequences that aligned in rhizomatous *Th. intermedium* but had no BLAST hits in nonrhizomatous *S. bicolor* were selected as candidates for rhizome‐related function.

A rhizome‐specific assembly for *Th. intermedium* was also generated in this study (NCBISequence Read Archive SRX3529031) and used to further reduce candidates. The assembly was aligned to the *Th. intermedium* protein BLAST database with a BLASTX search, identifying the sequences from rhizomes in the full *Th. intermedium* gene model set. The *Th. intermedium* predicted peptides declared present in rhizomes were then intersected with the *S. halepense* queries that had a *Th. intermedium* BLAST hit but not a hit from *S. bicolor*. The sequences that resulted are likely related to rhizome function, as they were expressed in *Th. intermedium* and *S. halepense* rhizomes, but do not appear in the genome of a closely related annual grain, *S. bicolor*.

## 3 RESULTS

### 3.1 Assembly

Johnsongrass rhizome RNA was extracted, sequenced, and trimmed to yield 283,167,847 paired reads in 72.1 gigabases with an average Phred score > 30 (NCBI Sequence Read Archive SRX3427471). The reads were assembled de novo in Trinity to produce 196,266 contigs. Using TransDecoder (version 3.0.1), 141,176 of the assembled contigs were identified as possible protein‐coding sequences. The proposed coding sequences have an average length of 825 base pairs (bp) and range from 297 to 15,174 bp. See Supporting Information Figure S3 for an illustration of the distribution of contig lengths for the filtered assembly. An N50 value of 1,071 bp indicates that the contigs of this length or longer make up 50% of the total assembly length in base pairs. Table 1 lists the statistics for the sequencing/assembly process as a whole.

**Table 1:** Quantitative overview of sequenced reads before and after cleaning, assembled contigs, and predicted coding sequences from TransDecoder

Sequencing and assembly statistics	
Total number of raw reads	286,173,708 × 2
Total number of cleaned reads	283,167,847 × 2
Total number of assembled contigs	196,226
Total number of assembled genes	93,708
Total number of protein coding sequences from TransDecoder	141,176
Mean length of coding sequences (bp)	825
Median length of coding sequences (bp)	570
N50 value of coding sequences (bp)	1,071
Range of coding sequence length (bp)	297–15,174
GC content	53.37%

### 3.2 Estimated abundance and assembly filtering

Quantification with kallisto identified 75,153 lowly expressed transcripts (FPKM < 1), which were removed from the assembly. After removing a further 1,576 sequences which had BLAST top hit results from nonplant organisms, 64,447 remained in a reduced assembly which had high likelihood to contain relevant functional information on the genetics of rhizome development in *S. halepense* (Supporting Information Data S3).

### 3.3 Annotation

A BLASTX‐fast search against the NCBI nonredundant protein database (NR) aligned 57,985 (90%) of the johnsongrass filtered assembly sequences with at least one hit (e‐value < 10^−20^). GO terms associated with BLAST hits were then mapped to 49,368 (76%) of the query sequences. Lastly, functional annotations were found for 46,792 (73%) of the sequences (Table 2).

**Table 2:** Results from the Blast2GO annotation suite

Blast2G0 statistics	
Sequences with BLASTX hits	57,985 (90%)
Mapped sequences	49,368 (76%)
Annotated sequences	46,792 (73%)
Gene ontology (GO)	
GO terms mapped	322,100
Biological processes (BP)	104,705
Molecular functions (MF)	125,645
Cellular components (CC)	91,750
10 most‐mapped GO terms	
Membrane (CC)	17,252
Integral component of membrane (CC)	16,388
ATP binding (MF)	7,651
Plastid (CC)	6,831
Nucleotide binding (MF)	6,816
Nucleus (CC)	6,463
Mitochondrion (CC)	6,201
Metal ion binding (MF)	6,157
Transferase activity (MF)	5,432
Hydrolase activity (MF)	4,705

*Sorghum bicolor* was the most prominently aligned species by far, receiving 51,544 of the top BLASTX hits (89%). The next highest amount of hits for a plant species was 3,953 (7%), from *Zea mays* (maize). A collection of fungi, bacteria, and animals accounted for 1,576 (2%) of the top alignments, but many of these may be from symbiotes or pathogens dwelling within the roots. See Supporting Information Figure S4 for the distribution of BLASTX top hits among the twenty most‐hit plant species.

A total of 322,100 gene ontology terms were mapped to the filtered johnsongrass contigs. These GO terms were split into three categories by their associations: 104,705 were for biological processes, 125,645 were for molecular functions, and 91,750 were for cellular components. The most‐mapped term was cellular “membrane” and was associated with 17,252 sequences. The results of gene ontology mapping and most common terms are listed in Table 2. Supporting Information Figure S5 depicts the distribution of GO level 1 terms over the three categories.

### 3.4 Comparative transcriptomics

**Figure 1:**
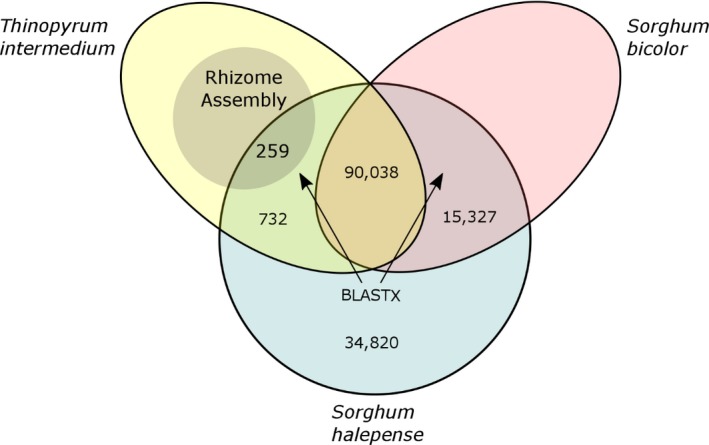
*Sorghum halepense* transcriptome comparison with *S. bicolor* and *Thinopyrum intermedium*. A Venn diagram depicts the distribution of *S*. *halepense* coding sequences as they have BLASTX hits against *Th. intermedium*,* S. bicolor*, both, or neither. An assembly of rhizome RNA from *Th. intermedium* was cross‐listed with the *S. halepense* sequences that had BLASTX hits from *Th. intermedium* but not *S. bicolor*. All quantities refer to unique *S. halepense* contigs

Of the 141,176 johnsongrass coding sequences, only 991 of the johnsongrass contigs had a BLASTX hit from rhizomatous *Th. intermedium* and no hit from annual *S. bicolor*. Of these selected contigs, 259 had the same BLASTX hit from *Th. intermedium* as a sequence from the *Th. intermedium* rhizome assembly. After removing sequences which may be lowly expressed or from nonplant organisms, 98 transcripts remained, which corresponded with 58 unique, putative peptide sequences from the *Th. intermedium* reference genome (Supporting Information Data S4 and S6, respectively). Figure 1 depicts the BLASTX comparisons of the johnsongrass contigs. Table 3 lists the specific results of these searches, and Supporting Information Data S5 lists the Blast2GO annotation results for the 98 candidate sequences grouped by function.

**Table 3:** Overview of BLASTX comparisons of johnsongrass contigs with *Sorghum bicolor*,* Thinopyrum intermedium*, and a rhizome assembly from *Th. intermedium*

BLASTX comparisons results	
Total number of *S. halepense* query sequences	141,176
Sequences with > 1 hit from *S. bicolor*	105,365
Sequences with > 1 hit from *Th. intermedium*	91,029
Sequences with > 1 hit from both organisms	90,038
Sequences with no hits	34,820
Sequences with > 1 hit from only *S. bicolor*	15,327
Sequences with > 1 hit from only *Th. intermedium*	732
Sequences with > 1 hit from only *Th. intermedium* and in the rhizome assembly	259
Sequences from above 259 with an FPKM value > 1	98

## 4 DISCUSSION/CONCLUSION

This study was meant to be an exploration of the rhizome transcriptome in a nonmodel organism. The lack of sample replication and of a reference genome or transcriptome have, however, led to some limitations in analysis.

Modern sequencing and assembly techniques were used, but as no reference exists for alignment methods, a de novo assembler, Trinity, was chosen. RNA‐sequencing and de novo assemblers tend produce to very large amounts of assembled transcripts, many of which are lowly expressed gene isoforms. To make the transcript set more likely to be biologically relevant, all lowly expressed sequences were removed, even though some of these still may have been relevant. Furthermore, as only a single tissue sample was sequenced, the calculated expression values have unverified accuracy.

Using presence/absence analysis via BLASTX searches against a *Th. intermedium* rhizome assembly and other genomes, 98 sufficiently expressed *S. halepense* sequences were selected as potentially related to rhizome behavior. This comparison method is dependent on BLASTX search parameters and a stringent e‐value < 10^−20^, so some sequences without or with lower quality matches may also have been relevant.

In any case, the functions of the 98 selected *S. halepense* sequences are very probably related to rhizomes. In Supporting Information Data S5, the 98 contigs are in groups of similar functions. The largest groupings of contigs are based on their relation to retrotransposons, mitochondrial DNA, cell membrane, and an F‐box protein. These are not obviously associated with rhizome function, although F‐box proteins are known to regulate signal transduction and will affect plant growth (Peng et al., 2012). The transcript with the largest expression value by far is in the ZIM (zinc finger inflorescence meristem) family motif. ZIM domain proteins are part of the signaling pathway of jasmonic acid, a hormone involved in plant growth, development, and responses to environmental stress (Liu et al., 2017; Major et al., 2017). The second most expressed transcript in the set is annotated as a member of the Armadillo (ARM)‐repeat superfamily. ARM proteins have shown differential expression under abiotic stress and in developmental conditions (Sharma et al., 2014). That these two sequences are highly expressed in harvested rhizomes is not surprising.

To gain more insight into the annotations of the rhizome‐specific contigs, GO enrichment analysis was performed. With the integrated FatiGO package within Blast2GO (Al‐Shahrour, Diaz‐Uriarte, & Dopazo, 2004), a Fisher's exact test was used to compare the GO terms mapped to the 98 rhizome‐specific contigs against those mapped to the johnsongrass filtered assembly (no lowly expressed sequences and no probable contaminants). The two sets are from the same tissue sample, meaning over‐ or underexpression should be due to the selection of rhizome‐specific sequences. A total of 12 terms were found to be overexpressed and 6 to be under‐expressed. The overexpressed terms included karyogamy and a variety of DNA processes and activities, which may be linked to cell division and coincide with rhizome behavior. The rest of the terms were for processes and activities of vitamin B6, which may be linked to development and nitrogen metabolism (Colinas et al., 2016). The results of the Fisher's exact test are listed in Supporting Information Data S7.

The 58 peptide sequences from *Th. intermedium* that corresponded with the rhizome‐related *S. halepense* contigs were run through the CD‐Search Tool (Marchler‐Bauer & Bryant, 2004). A total of 300 conserved domains were aligned with 40 of the sequences. The alignment results, as well as the *S. halepense* contig IDs associated with each protein sequence are listed in Supporting Information Data S8. Broadly, the alignments have a theme of DNA modification, including some transcription factors, transposases, zinc fingers, and variety of other DNA/RNA binding domains. Transcription factors such as WRKY4 (transcript TRINITY_DN40459_c3_g1_i5_g.83411_m.83411) are notable in this study, as a main anticipated difference between *S. halepense* and recently rhizomatous *S. bicolor* would be in expression levels. In Hu et al., 2011; there were 24 transcription factor genes upregulated in rhizome tips, some of which also encoded WRKY domains. Another upregulated gene from the same study encoded kinase family proteins, just as several transcripts in the rhizome‐specific johnsongrass set do. The large range of functional categories for the rhizome‐related johnsongrass transcripts complies with the suggestion of Jang et al., 2006; —that members within gene families may specialize in an area such as the rhizome meristems. In fact, the putative lipid transfer protein used as an example in the previous study may also be present in the 98 rhizome‐specific contigs as TRINITY_DN33084_c0_g1_i2_g.31287_m.31287. Seven genes encoding such lipid transfer proteins were found to be upregulated in rhizome tips (Hu et al., 2011).

Also in Jang et al., 2006, developmental and environmental responses specific to rhizomes were cited as reasoning for numerous highly expressed genes affecting hormones, signaling proteins, abiotic stimuli, and development. Highly expressed contigs in the 98 rhizome‐specific set such as those of the ZIM family, ARM repeats, or F‐box annotations fit this description well.

The identification of genes essential to perenniality remains an important, and yet, elusive biological problem, especially in efforts to perennialize grain crops and enhance the sustainability of food production. Recent efforts to gather expression data on rhizomatous tissue are providing important lists of candidate genes to accelerate the process of uncovering the biological pathways related to perenniality. This study provides an important step in the process, as well as providing a high‐confidence set of contigs (64,447 predicted contigs) for the, as yet, unsequenced *S. halepense*.

The 98 candidate sequences will now be the focus of further bioinformatics analysis (e.g., protein functional prediction, enhanced annotation, and BLASTing). In particular, promising candidates may become the target of genetic modification (e.g., knockout; overexpression) experiments to elucidate genes critically part of grain crop perenniality.

Quantitative trait loci associated with rhizome function may be used to improve breeding practices for perennial traits in a Sorghum hybrid. This study has produced a comprehensive dataset which facilitates further research with *S  halepense* and other perennial grains.

## DATA ACCESSION

The johnsongrass dataset has been registered with NCBI as a BioProject (PRJNA417857). The raw read files are available in the Sequence Read Archive (SRP125786), and this Transcriptome Shotgun Assembly project has been deposited at DDBJ/EMBL/GenBank under the accession GGDZ00000000. The version described in this article is the first version, GGDZ01000000.

The *Th. intermedium* dataset has been registered with NCBI as a BioProject (PRJNA428355). The raw read files are available in the Sequence Read Archive (SRP127982), and this Transcriptome Shotgun Assembly project has been deposited at DDBJ/EMBL/GenBank under the accession GGEN00000000. The version described in this article is the first version, GGEN01000000.

## AUTHOR CONTRIBUTIONS

NLT, KMD, and JP conceived and supervised this study. NR and MH extracted *S. halepense* RNA from the rhizomes harvested by LDH and processed by JP. KMD extracted from the *Th. intermedium* rhizomes and assembled both rhizome assemblies after sequencing. SL, MA, NLT, and NR performed the annotation and analysis. NR wrote the manuscript with contributions from all other authors.
