# Supplementary material for: Transcriptome assembly and annotation of johnsongrass (Sorghum halepense) rhizomes identify candidate rhizome‐specific genes
Source: Plant Direct. 2018 Jun 19;2(6):e00065. doi: 10.1002/pld3.65 (PMC6508516; doi:10.1002/pld3.65)
Supplement: Supplementary file 1 [file PLD3-2-e00065-s001.pdf]

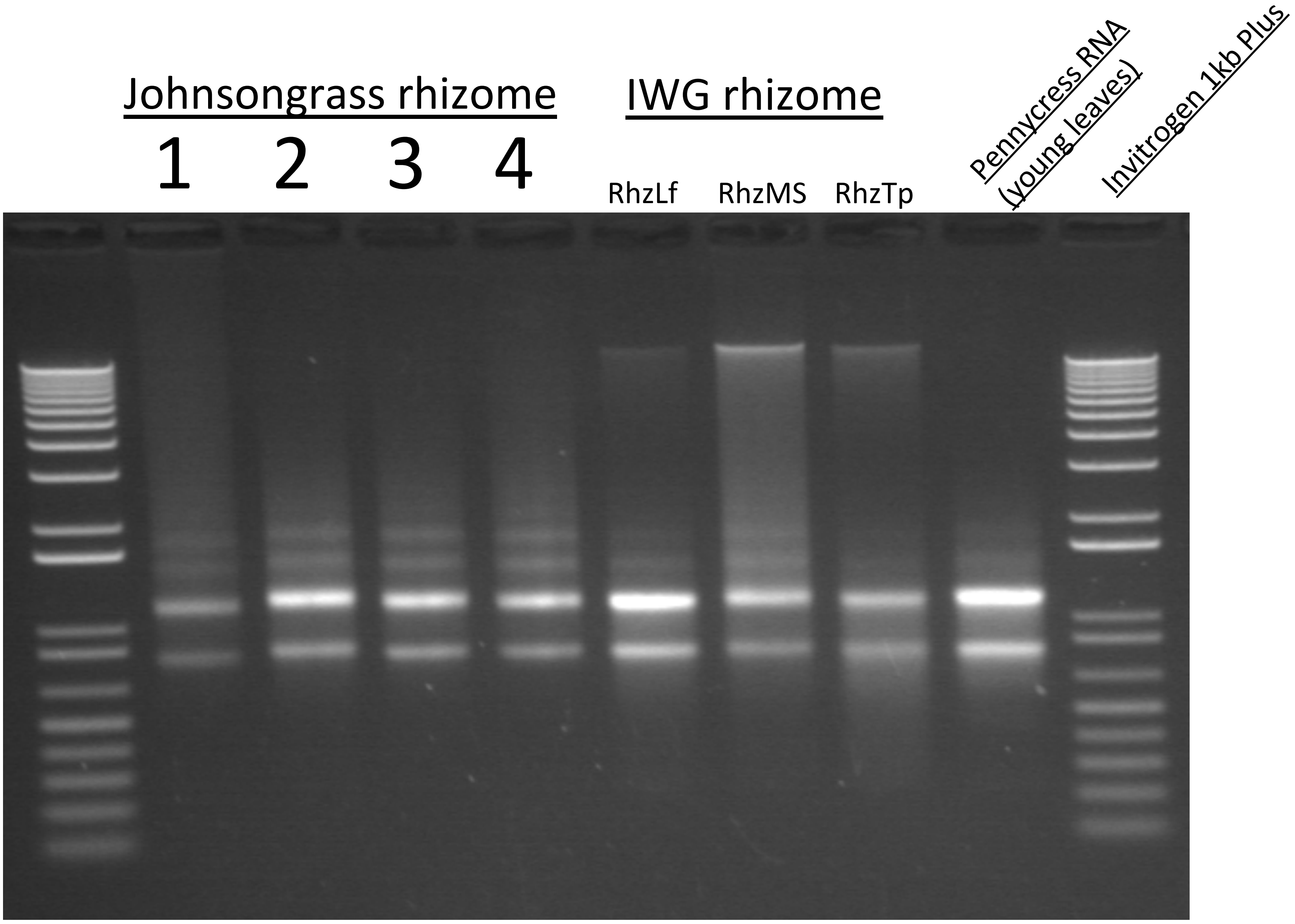
Johnsongrass rhizome
1
2
3
4
IWG rhizome
RhzLf
RhzMS
RhzTp
Pennycress RNA
(young leaves)
Invitrogen 1kb Plus
