# Supplementary material for: Transcriptome assembly and annotation of johnsongrass (Sorghum halepense) rhizomes identify candidate rhizome‐specific genes
Source: Plant Direct. 2018 Jun 19;2(6):e00065. doi: 10.1002/pld3.65 (PMC6508516; doi:10.1002/pld3.65)

*Supplemental Figure 2: Distribution of FPKM values for the johnsongrass assembly sequences. The values below 1 seem to form a skewed gaussian distribution, which has a tail that extends to beyond 11,000. For the sake of recognizing the behavior below 1, all values between 20 and 11,437 have been accumulated into a single bar, although the distribution continues in the same manner from 1 all the way to 11,437. This behavior below 1 may indicate a baseline level of transcriptional noise, such as the low-level transcription of non-gene portions of the genome. Using a cutoff of FPKM < 1 gives the remaining sequences higher confidence of being biologically relevant.*


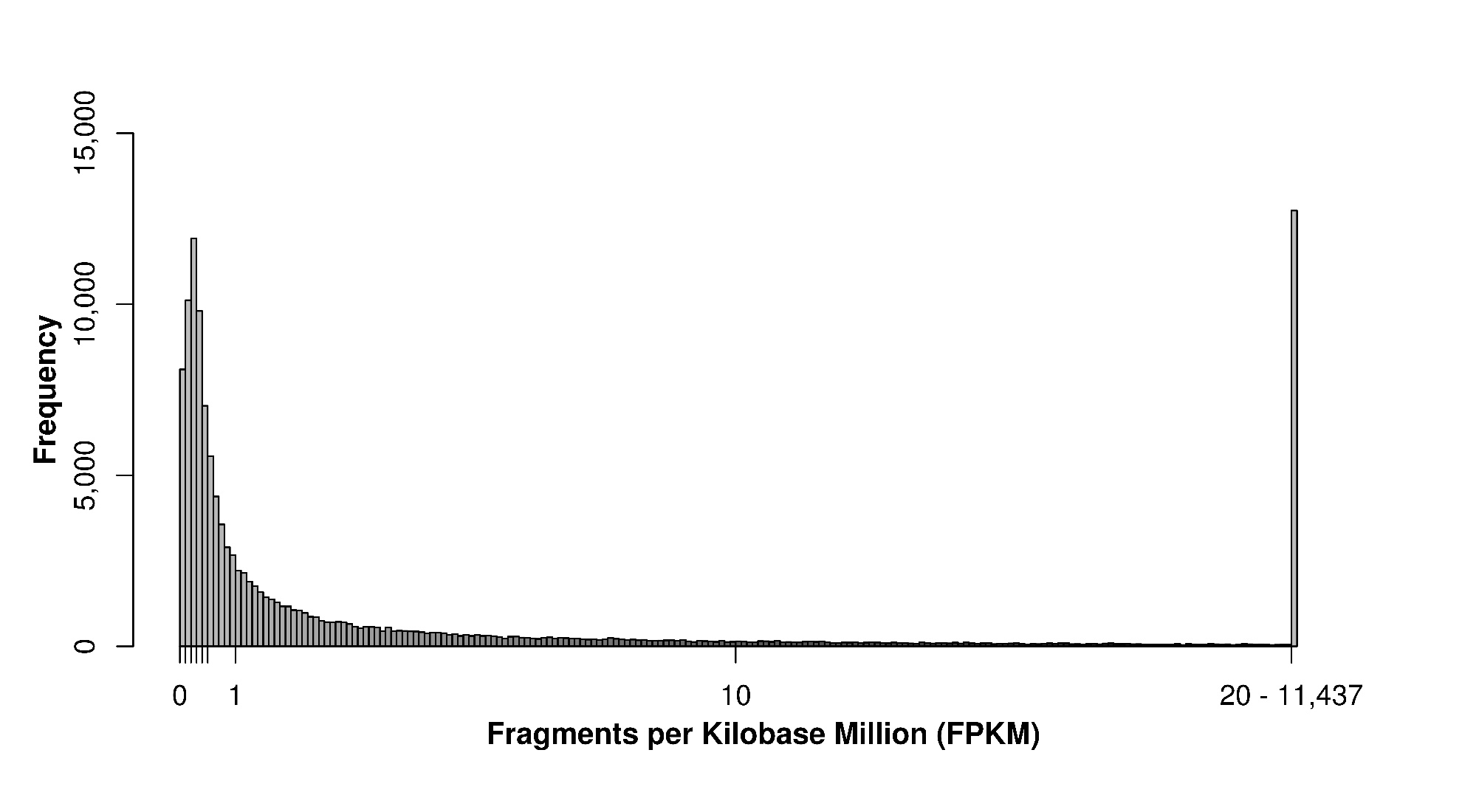

Supplement: Supplementary file 2 [file PLD3-2-e00065-s002.docx]
