# Supplementary material for: Transcriptome assembly and annotation of johnsongrass (Sorghum halepense) rhizomes identify candidate rhizome‐specific genes
Source: Plant Direct. 2018 Jun 19;2(6):e00065. doi: 10.1002/pld3.65 (PMC6508516; doi:10.1002/pld3.65)

*Supplemental Figure 3: Distribution of base-pair lengths for contiguous sequences generated via Trinity assembly pipeline and predicted to be coding sequences in TransDecoder.*


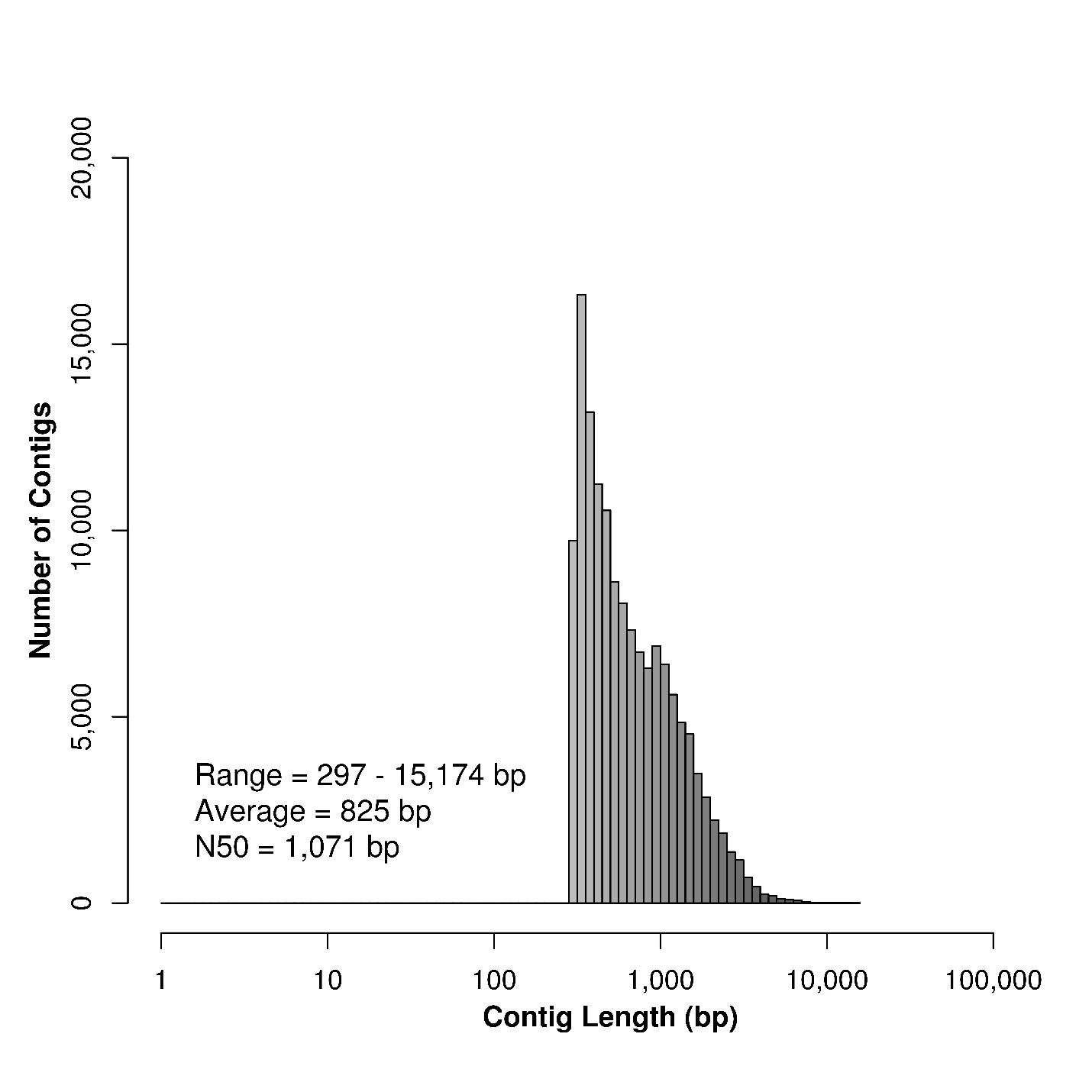

Supplement: Supplementary file 3 [file PLD3-2-e00065-s003.docx]
