# Supplementary material for: Transcriptome assembly and annotation of johnsongrass (Sorghum halepense) rhizomes identify candidate rhizome‐specific genes
Source: Plant Direct. 2018 Jun 19;2(6):e00065. doi: 10.1002/pld3.65 (PMC6508516; doi:10.1002/pld3.65)

*Supplemental Figure 4: Species distribution of BLASTX top hits from the TransDecoder predicted coding sequences queried against the NCBI non-redundant protein database (NR). Only members of kingdom Plantae are included.*


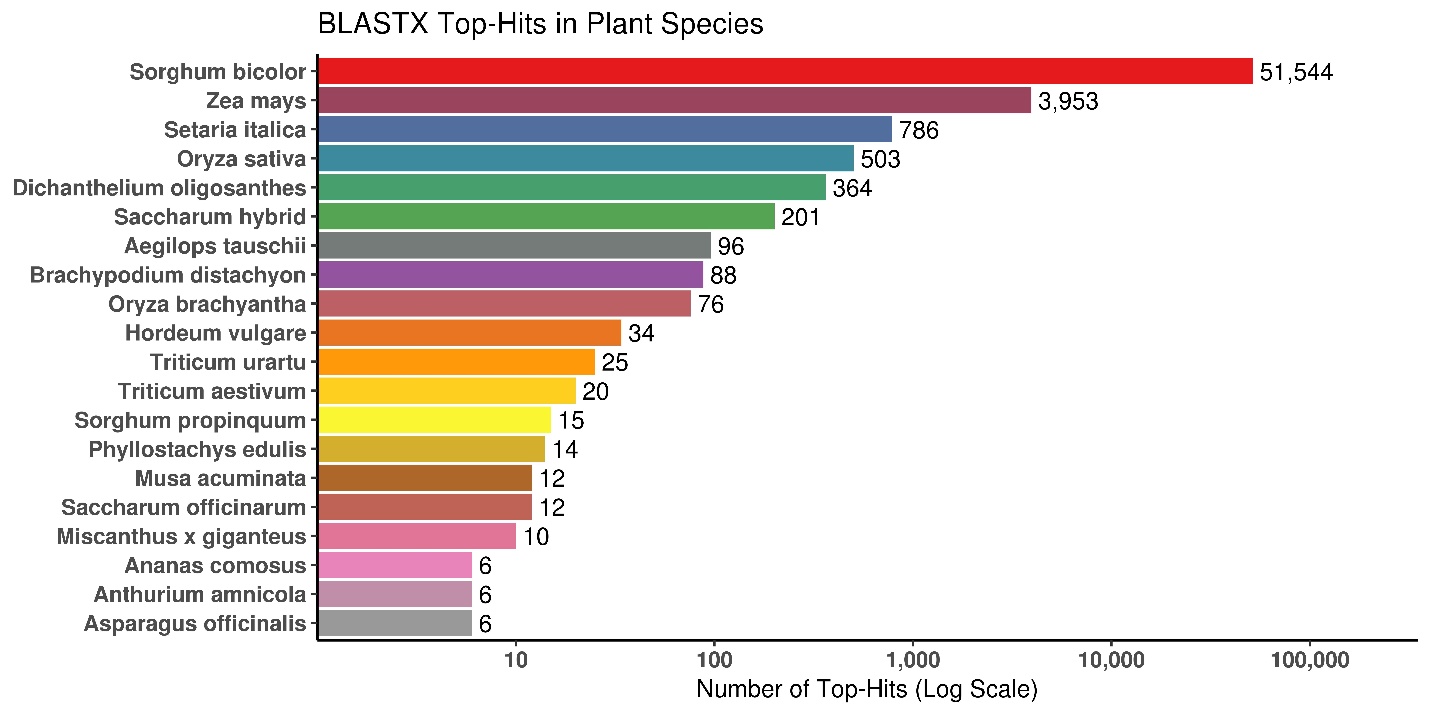

Supplement: Supplementary file 4 [file PLD3-2-e00065-s004.docx]
