# Supplementary material for: Transcriptome assembly and annotation of johnsongrass (Sorghum halepense) rhizomes identify candidate rhizome‐specific genes
Source: Plant Direct. 2018 Jun 19;2(6):e00065. doi: 10.1002/pld3.65 (PMC6508516; doi:10.1002/pld3.65)

*Supplemental Figure 5: Distribution of GO level 1 terms by categories Biological Process (BP), Molecular Function (MF), and Cellular Component (CC). Only the terms mapped to sequences of the filtered assembly (without lowly expressed or non-plant BLASTX results) are depicted. The top 5 mapped terms are listed for each category, with all remaining terms in that category grouped into “other”.*


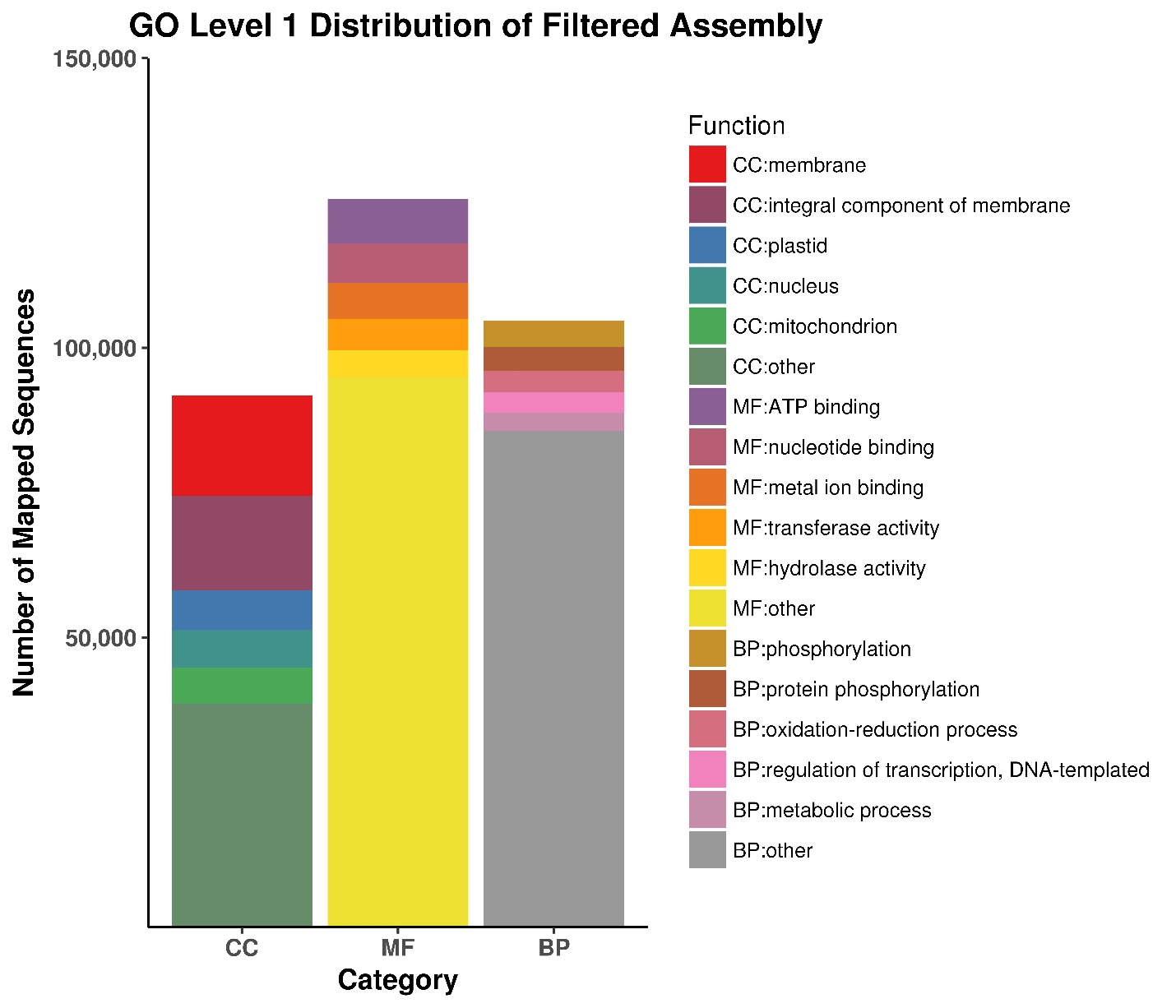

Supplement: Supplementary file 5 [file PLD3-2-e00065-s005.docx]
