# Supplementary material for: Transcriptome assembly and annotation of johnsongrass (Sorghum halepense) rhizomes identify candidate rhizome‐specific genes
Source: Plant Direct. 2018 Jun 19;2(6):e00065. doi: 10.1002/pld3.65 (PMC6508516; doi:10.1002/pld3.65)
Supplement: Supplementary file 14 [file PLD3-2-e00065-s014.pdf]

Reviewer Responses 02/02/2018

Reviewer #2:

This submission describes the rhizome transcriptome of Sorghum halepense, commonly known as johnsongrass. The authors describe sequencing and assembly of the transcriptome. The most interesting part of the analysis is the profiling of the predicted proteins identified in their johnsongrass transcriptomes vs. other genomic data in order determine proteins specific to rhizomous plants. This investigation identified 259 genes, for which the authors provide limited functional annotation. The manuscript is appropriately concise and generally well written, although some clarification in a number of places would be helpful, as summarized below.

Major points
1) the comparative analysis is quite minimalist, and would likely benefit from being more inclusive of genomes from other organisms that develop or do not develop rhizomes. This would serve to increase the resolution of their analysis. Minimally, the authors should comparatively analyses/discuss in the context of the findings of Hu et al 2011 (Identification of rhizome-specific genes by genome-wide differential expression Analysis in Oryza longistaminata), which the authors have missed.

I wasn't able to find the actual sequences referenced in the paper, but added discussion of our findings in light of their study.

2) It's possible that I missed it, but I've read through the relevant sections multiple times and it is still not clear to me how the authors arrived at 991 (L179). Is this number represented by the brown circle in Figure 4? The intersects of the Venn would suggest not, but if not what is this circle labeled Rhizome Assembly?

I've adjusted Table 3, which previously listed (erroneously) 732 as the number of sequences with blast hits from only *Th. intermedium.* It now reads 991. This may have led to some of your confusion.

For further clarification:
There are 991 sequences which received a BLASTX hit from the full draft genome of *Th. Intermedium* but did <u>not</u> receive a hit from *S. bicolor*. Thus, these sequences were aligned with a rhizomatous grass and not aligned to a closely related annual. The brown circle in the figure represents the assembly made from just *Th. Intermedium* rhizomes. The 991 sequences were intersected with the rhizome-specific assembly to end with 259 sequences. In Figure 4 (now Figure 1), the total sequences within the *Th. Intermedium* bubble and not the *S. bicolor* bubble is 991.

3) There is no discussion as to the extent of expression of any gene. Do the authors intend to publish this elsewhere?

Quantification estimates are now included in the manuscript:
I ran kallisto on the trimmed read set to find estimated abundances for the assembly transcripts. This is a single sequenced sample from an organism without a reference transcriptome, so the accuracy of expression values is limited. The top 20 most expressed sequences and their descriptions/annotations are listed in Supplementary File 1. I used a threshold of FPKM < 1 to remove lowly expressed transcripts

that are likely to be noise from background transcription. This may be a stringent threshold, but the FPKM values form a skewed gaussian distribution situated below 1 with a long tail extending to >11,000. This behavior could indicate a baseline level of transcriptional noise, such as the low-level transcription of non-gene portions of the genome. Using a cutoff of FPKM < 1 gives the remaining sequences higher confidence of being biologically relevant.

Expression of the rhizome candidates is included in Supplementary File 5.

4) Functional annotation of the 259 predicted rhizome-specific genes is minimal. I strongly encourage more analysis of the proteins encoded by these genes. Non-exhaustive suggestions might include conserved domains, gene co-expression analysis (also see above), any investigations relating to these proteins elsewhere in the literature etc.

The rhizome-specific set has been further analyzed with conserved domains and its annotation compared with two other rhizome-based studies.

Minor points

1) I am not sure that Figures 1-3 are sufficiently significant to warrant placement in the main article, and suggest moving to Supplemental Information. It's not clear from the title of Figure 3 which group of genes this is. Also the authors may wish to consider splitting Table 3 into two Tables; it is presently a bit confusing.

I have made Figures 1-3 to now be supplemental. I also made a new Figure 3 (now Supp. Figure 5), which depicts the distribution of GO level 1, not level 2. This graph is easier to read and level 1 annotations may be more appropriate, as the graph is simply meant to provide an overview of the GO mappings to assembly contigs. I also added a more descriptive title.
I removed the second part of Table 3. Instead, GO enrichment analysis results for the 98 rhizome-specific contigs are listed in Supplementary File 9.

2) L75-77 this statement should be qualified as to how the transcriptomes might help. Do the authors mean by providing potential engineering targets?

The main benefits of the transcriptomes would be to A) assist breeding of perennial grains (e.g. with marker-assisted selection based on genes or loci found to be related to rhizome function) and B) provide a novel transcriptome for a non-model organism. I mention these motivations in the two paragraphs below L75-77 respectively. I altered L75-77 to be more succinct, but could include more possible benefits if these are not sufficient.

3) L84 what is meant by connected? Does this mean mentioned?

I edited L84 to clarify the type of connections the cited studies draw.

4) In light of major comment 4 above it seems excessive to describe these are well-annotated (L86)

Removed the descriptor “well-annotated”.

5) L106 were any measurements taken for RNA quality? Eg a RIN from a Bioanalyzer? If not, it might be useful to include the gel image as a Supplemental Figure

Added a gel image of the samples compared with standards as Supplemental Figure 1.

6) L110 why were duplicated reads removed? Unless I'm missing something, this would have the effect of removing quantitativeness from the transcriptomes?

I was incorrect. Duplicates were not removed at the bbduk step. They are, instead, normalized going into the assembly process by Trinity to improve performance. To produce an abundance estimates for the contigs with kallisto however, I used the non-normalized trimmed read set (after bbduk, before Trinity).

7) Sequencing statistics should be included for the johnsongrass sequencing If they are also included for T. intermedium (L114-115)

Added the sequencing stats for johnsongrass. They should be the same now.

8) L165 suggest removing "in NR" and providing the threshold cutoff used

Edited the manuscript per your suggestion.

9) L170-171 were these presumed contaminants removed from the comparative analysis?

All 141,176 coding sequences were included in the comparative analysis, but no potential contaminants were found in the “rhizome-specific” contigs (as these contigs were all aligned with plant species). Now lowly expressed sequences have been removed.

10) The assembled transcripts should be further clustered into super contigs to remove the redundant assembled product. The overall number of contig set (~141 000) is still too high.

After removing lowly expressed and potential contaminant sequences, the remaining high confidence set is at 64,447.

11) The GO analysis of Figure 3 should be conducted by the GO enrichment analysis instead of the actual number of GO term hits.

I made a new Figure 3 with level 1 annotations (see Minor Point #1) and also did enrichment analysis. I performed a Fisher’s Exact Test, using the 98 “rhizome-specific” johnsongrass assembly sequences as a test set – a group of genes/fragments of genes that may be expressed differently than the rest of the transcriptome. I used the high confidence sequences (no lowly expressed, no non-plants) as a reference set. The results are listed in Supplementary File 9.

12) The x-axis scale can be in logarithm in the Figure 1 for better recognize.

I revised the figure to have the x-axis in log10 scale.

Reviewer #3:

This manuscript is a resource paper providing an annotated rhizome transcriptome set for S. halepense (Johnosongrass). It also provides the rhizome transcriptome for Thinopyrum intermedium. Comparing the two rhizome transcriptomes with the non-rhizome S. bicolor produced a set of 259 sequences that may be associated with rhizome development.

The writing of the manuscript is relatively clear, and may be sufficient for a resource paper, but not for a research paper. It provides no discussion of the results. There is no discussion on how their results compare with other published rhizome transcriptome results. They provide a URL that is suppose to have the results, but it is a link to a software page.

The discussion has been appended to include more in-depth consideration of the annotations and comparisons with previous studies.
Supplementary data has been submitted to the journal.

Methods:
1. Library Construction and Sequencing: Is the first paragraph discussing Johnsongrass? The total Gibabases is given for Th. intermedium but not Johnsongrass. It sounds like Johnsongrass was trimmed but does not say if Th. intermedium was trimmed.

Yes, it is referring to the johnsongrass RNA samples. I added mention of the organism. I also added the total Gigabases for Johnsongrass. Johnsongrass and Th. intermedium were both trimmed, I appended the procedure for the Th. intermedium set to include this.

2. Assembly: Were both sets of reads assembled the same?

Yes, the same trimming, filtering, and Trinity assembly parameters were used.

3. Comparative Transcriptomes: The use of the full Tr. intermedium transcript set with subsequent filtering with the rhizome set sounds very ad hoc. Explain the advantage of this pipeline. Was the only criteria a blast hit of e-value<1E-20?

The full Th. intermedium set is a draft genome, giving these transcripts higher confidence than the rhizome set and also providing their putative peptide sequences. To compare the two rhizome assemblies directly (via BLAST) would have required a nucleotide BLAST search or making a set of predicted peptides for one of the two sets, either option relying on single-sample RNA sequencing results. We aligned both the johnsongrass assembly and the Th. intermedium rhizome set to the full Th. intermedium full-genome protein database. This means that any transcripts matched between the Th. intermedium and johnsongrass rhizome assemblies were aligned with the same Th. intermedium genome peptide sequence, which is more likely to be real than either assembly transcript.

The BLASTX search had the constraint of e-value < 10^{-20} as well as the default command-line parameters: https://www.ncbi.nlm.nih.gov/books/NBK279684/#appendices.Options_for_the_commandline_a (as per table C4)

Verification of the results:
I was puzzled that there were 141,176 sequences from the Johnsongrass rhizome set. So I downloaded the S. halepense from Genbank and assembled the contigs using a CAP-based assembler with the strict restrictions of 100% identity and minimum of 200bp overlap, which resulted in 91618 contigs. In fact, over 28000 appear to be exact duplicates (one contig of contigs has 50 duplicates).

I filtered with expression levels and BLAST results to produce a set of 64,447 sequences that are most likely to be biologically relevant. There are still several thousand isoforms in this set that may appear as exact duplicates, but Trinity should have based these on the reads it was provided.

I compared the two translated rhizome sets with the Genbank S.bicolor proteins using a requirement of 90% overlap and 90% identity, and got over a 1000 rhizome specific sets (at least one contig from each rhizome set and none from S.bicolor). Is there any prediction on how many rhizome specific transcripts would be expected?

This is a limitation of the study. Our comparison method (BLASTX searches) may be more stringent than others and we are not aware of an estimate for the amount of transcripts found to be rhizome specific. This limitation is now mentioned in the discussion.

Reviewer #5:

Please see the following comments. I encourage you to keep working on this project and address the issues in the current version of the manuscript.

This manuscript opens with a discussion of rhizomes and their potential benefits for agriculture. This introduction does a good job establishing the potential environmental benefit of perennial crops over annual crops, especially for nitrogen runoff and carbon sequestration. It establishes the context for the results presented later in the manuscript, and the role that these results can play in aiding the development of perennial hybrid of crop sorghum. Finally, it references previous work to identify RNAs involved in rhizome functions, and mentions the limitations of those studies. The new RNAseq data generated for this manuscript can address those limitations, and bring the field closer to the goals presented in the introduction.
Unfortunately, the rest of the analysis is not comprehensive enough to address those limitations. The transcriptomes are assembled using Trinity, with coding sequences predicted using TransDecoder. While this is a valid de novo assembly workflow especially for preliminary analysis, both of these tools can greatly over-estimate the number of contigs present in the assembly, leading to a high degree of artificial redundancy in the assembly (see e.g. the supplemental data for Peng, et al. (2016) "A novel codon-based de Bruijn graph algorithm for gene construction from unassembled transcriptomes," Genome Biology, 17:232). This redundancy can be addressed with additional verification of the assembled contigs. At a minimum using gene quantification based on expectation maximization (e.g. Kallisto or Salmon) to check estimated expression can find at least a subset of artificially duplicated genes. Since these further analyses were not performed, it is questionable how many of the genes presented in the results are real.

Performed quantification estimation per your suggestion and also filtered via top BLAST results to produce a set of 64,447 higher-confidence contigs.

Additionally, many of the points raised in the introduction and the annotations performed are not addressed in the discussion. While the introduction mentions the previous work on RNA in rhizomes, the relationship between the new results and previous work is never mentioned. The work done on annotating the assembled transcriptome using BlastX and Blast2Go is mentioned in the abstract, introduction, and results, including two tables and two figures, but the significance of those annotations are never discussed. Instead, most of the discussion focuses on the future work that can be performed on the generated data.

Annotation results are now discussed and parallels with previous studies are drawn.

The RNAseq data generated in this study and their initial work on assembling and annotating the data is certainly important enough to justify a publication in some form. However, considering that only preliminary analyses are included and they are not actually discussed, this manuscript cannot be accepted as a full research article. My suggestion is to revise this manuscript much shorter and more concise, and submit it as a data announcement paper elsewhere (unless Plant Direct accepts data announcement papers). Conversely, the current manuscript could form the core of a full research article if it is extensively revised including additional analyses and more in-depth verification and discussion of the results.

The manuscript has been heavily revised to now include further analyses and a more rigorous discussion.

As a final note, Figures 1 and 3 appear to have issues with the resolution. Figure 1 appears very blurry when printed, and Figure 3 is nearly illegible. Regardless of the revisions performed, these figures will need to be saved at a higher resolution or using scalable graphics.

Figure 1-3 are now supplemental figures and at a higher resolution. I made a new Figure 3 (now Supp. Figure 5), which is more readable and depicts the distribution of GO level 1, not level 2.

Reviewer #6 :

This work by Ryder, et al. is a preliminary release of data for a Sorghum rhizome project. The sequence processing and assembly workflow uses solid, current methods and software and everything is versioned and cited appropriately. The study brings novelty in rhizome formation and development, but is poorly discussed. The discussion was limited to the results of sequencing. The contributions to rhizome biology is not approached in the manuscript. For example: what are the specific genes for rhizomes? What are their functions? Biologically discussion is required.

The discussion section now looks further into annotation results and makes comparisons with past studies.

Also, on Figure 3 we can not read the y axis.

I made a new Figure 3 (now Supp. Figure 5), which is more readable and depicts the distribution of GO level 1, not level 2.

I have a few small suggestions below, but would support the publication of
the manuscript largely in its present form.

Minor comments:
l.66 and in Table 1- italics missing on taxonomic name

Added italics per your suggestion.

It would be nice to see the BLAST2GO results posted to a more stable URL
stable than just a long university departmental website.
This makes the longevity of the URL contingent on the University's webmaster.
Do Phytyozome or other data services accommodate this?
or a grass-specific database FTP site?

Supplementary data has been submitted to the journal.
